# Deep roots delay flowering and relax the impact of floral traits and associated pollinators in steppe plants

**DOI:** 10.1371/journal.pone.0173921

**Published:** 2017-03-16

**Authors:** Rachda Berrached, Leila Kadik, Hocine Ait Mouheb, Andreas Prinzing

**Affiliations:** 1 Laboratory of Ecology and Environment, Faculty of Biological Sciences, University of Sciences and Technology Houari Boumediene, Bab Ezzouar, Algiers, Algeria; 2 Research Unit “Ecosystèmes Biodiversité, Evolution”, Centre National de la Recherche Scientifique, Université Rennes 1, Campus de Beaulieu, Bât 14 A, Rennes, France; Estacion Experimental de Zonas Aridas, SPAIN

## Abstract

Strong seasonality in abiotic harshness and pollinator availability shape the reproductive success of plants. Plant species can avoid or can tolerate harsh abiotic conditions and can attract different pollinators, but it remains unknown (i) which of these capacities is most important for flowering phenology, (ii) whether tolerance/avoidance of abiotic harshness reinforces or relaxes the phenological differentiation of species attracting different pollinators. We assembled possibly the first functional trait database for a North African steppe covering 104 species. We inferred avoidance of harshness (drought) from dormancy, i.e. annual life-span and seed size. We inferred tolerance or resistance to harshness from small specific leaf area, small stature, deep roots and high dry matter content. We inferred the type of pollinators attracted from floral colour, shape and depth. We found that avoidance traits did not affect flowering phenology, and among tolerance traits only deep roots had an effect by delaying flowering. Flower colour (red or purple), and occasionally flower depth, delayed flowering. Dish, gullet and flag shape accelerated flowering. Interactive effects however were at least as important, inversing the mentioned relationship between floral characters and flowering phenology. Specifically, among drought-tolerant deep-rooted species, flowering phenologies converged among floral types attracting different pollinators, without becoming less variable overall. Direct and interactive effects of root depth and floral traits explained at least 45% of the variance in flowering phenology. Also, conclusions on interactive effects were highly consistent with and without including information on family identity or outliers. Overall, roots and floral syndromes strongly control flowering phenology, while many other traits do not. Surprisingly, floral syndromes and the related pollinators appear to constrain phenology mainly in shallow-rooted, abiotically little tolerant species. Lack of abiotic tolerance might hence constrain accessible resources and thereby impose a stronger synchronization with biotic partners such as pollinators.

## Introduction

Flowering phenology is one of the most studied traits in plants as it can strongly influence their reproductive success [1–3]. Flowering phenology obviously is constrained by the physiological constraints imposed by different seasonal environments. Seasons that are too harsh to survive are obviously too harsh to flower [4]. Among communities, flowering phenology is driven by functional traits that permit plants to sustain environmental stress and disturbance [5]. Flowering phenology is also constrained by pollinator availability in different environments [6]. Physiological constraints and pollinators do not only vary among environments, but also among species responding differently to abiotic harshness or attracting different pollinator types within the same environment. However the question which of these factors—response to abiotic harshness or attractiveness to pollinator types- most strongly affects flowering phenology within a given environment has received limited attention so far [7, 8]. In particular any interactive effects among both factors appear to be unknown.

Drought is a major type of abiotic harshness that might constrain flowering phenology–in particular in plants that avoid drought through dormancy. Seasonally dry environments are wide-spread across the globe and risk to further expand. Many species avoid drought by seasonal dormancy, especially annual herbs that complete their life cycle in just one single season, reproduce just before the onset of the dry season and persist dry season as dormant seeds [9]. Annual plants may hence be more restricted in flowering phenology to the favorable season [10].Perennial plants, in contrast, have roots or rhizomes permitting to store carbohydrates while above ground parts may be dormant or maintain some activity [2]. Perennials hence do not avoid drought and may flower later [11]. Moreover, larger seeds might be able to store large quantities of nutrients, providing seedlings a greater survival advantage during the long summer drought, whereas seedlings emerging from small seeds depend on environmental resources and are hence more affected by drought stress [12]. Thus, annual life form and possibly large seeds increase the ability of species to avoid drought over time and might accordingly constrain flowering.

Some plants do not avoid, but tolerate drought for instance due to morphological adaptations and may flower when others cannot. Multiple studies have shown that plants have evolved physiological and morphological strategies to reduce the impact of water limitation [10, 13, 14, 15]. Plants with deep roots can tolerate water-limited environments [14, 15], and are able to access deeper soil water [10] and to store significant quantities of water in their root tissues [16]. Consequently, phenological patterns of deep-rooted species might be independent of drought. Moreover, some plants can maintain active under drought by reducing the length of their shoots relative to the depth of their roots [17–19]. Also, small relative leaf surfaces and high dry matter contents might reduce transpiration, improve storage of carbon and permit leaves to maintain photosynthesis during dry periods [20]. This permits plants to flower even during the dry season [16] or just after the dry season [21]. Overall, species with deep roots, short shoots and small and heavy, sclerotized leaves may remain active during drier seasons and might flower later than other species.

Flowering phenology can also be highly constrained by plant-pollinator interactions [3, 22]. Many plant species evolved floral syndromes that attract particular pollinators [23, 24]. Such pollination syndromes, suites of phenotypic floral traits reflecting convergent adaptations of flowers to a particular type of flower visitors [25, 26], may structure ecological networks [27]. Thus, functional flower traits have evolved as response to mutualistic interactions [28]. Specifically, it has been shown that pollinators specialize on certain flower traits such as flower depth and floral reflectance [27], or flower shape (e.g., [29]). Long-tongued pollinators, for instance, feed preferentially at deep flowers and short-tongued pollinators on shallow flowers [6, 30]. Different pollinators, in turn, have different seasonal activities, i.e. pollinator phenologies [29, 31]. Differences in flower colour, shape and depth should hence correspond to different flowering phenologies.

A plant’s tolerance to abiotic harshness like drought, and the pollinators the plant attracts, may both affect flowering phenology [32] and even do so interactively. Here, we suggested two alternative hypotheses. First, we hypnotize that tolerance to drought stress might strengthen the temporal match between plants and pollinators. Plants remaining active during dry seasons may be more flexible to match flowering phenology to pollinator type. Simply, the temporal window during which the plant can develop flowers is larger than for plants that do not tolerate drought. Moreover, plants remaining active during dry seasons may face carbon stress rendering investment into nectar production [33, 34] and flowering more costly than during the wet season. Also, nectar volume may be water-limited [34] rendering flowering in the dry season more costly also in terms of water use. Thus, plants that are remaining active during drought should be under strong selection to optimize the match between flowering phenology and pollinator mutualists. Second, it might be the lack of drought tolerance that strengthens temporal match between plants and pollinator mutualists. A plant that faces a baseline water stress may need to optimize its investment of water and carbon into flowering, inducing a tradeoff between vegetative and reproductive growth [2] and hence limiting flowering phenology. Such optimization may be less crucial in a plant without such baseline stress. Given that flower traits correspond to pollination vectors [23–25], the relationship between flower traits and flowering phenology might hence be either strongest or weakest in plants showing adaptation to drought stress and remaining active during the dry season.

In this study, we investigate flowering phenology of plant species in a semiarid steppe ecosystem in Algeria. We ask the following questions: Does flowering phenology of a plant species depend on the species’ ability to avoid or tolerate water stress (as inferred from vegetative traits as outlined above)? Does flowering phenology depend on the plants’ interaction with pollinators (as inferred from flower traits as outlined above)? Does plant-pollinator interaction become stronger or weaker among plants that are stress-tolerant? To respond to these questions, we characterized flowering phenology and established probably the first comprehensive functional trait database for a North African steppe vegetation.

## Materials and methods

### Study area

The study was carried out at Djelfa Department, located approximately 300 km South of Algiers, Algeria. The study area is a grazing exclosure, managed by the High Commission for Development of the Steppe (HCDS). Grazing exclosures are a regeneration technique of steppe vegetation, and consist in protecting a degraded surface from any anthropogenic action [35]. Such grazing exclosures prevent anthropogenic disturbance of plants and their phenology notably in the permanent plots we installed.

The climate in this region is of semiarid Mediterranean type, characterized by a hot and dry summer and cold winter [36]. The average temperature is 15.4°C, ranging from 0.3°C in January to 33.7°C in July. Annual precipitation, measured over the last 39 years, is 318.5 mm; mainly in winter. The dry period is about six months from May to October. The vegetation is steppic dominated by the presence of *Artemisia herba alba* Asso. as the most abundant shrub species, and numerous herbaceous species such as *Helianthemum croceum* (Desf.) Pers., *Alyssum granatense* B. et R., *Centaurea involucrata* Desf., *Calendula aegyptiaca* Desf. and *Bromus rubens* L.

### Flowering phenology

Fifteen permanent plots of 10×10 m were randomly set up at the start of the fieldwork and used throughout the entire season for identification of flowering species and collection of specimens. Plots are set up in the way to cover the entire study area, and represent all vegetation types (note that vegetation mosaics were homogeneously distributed across the area). The installation of plots in the study area has required permission. A convention between the faculty of biological sciences and the High Commission for Development of the Steppe has been established. Furthermore, our field study did not involve endangered species and did not alter the vegetation. Flowering phenology was recorded twice a month in the main flowering season, from early March to late May, and once a month out of this season, 2015.

At each monitoring time, plants presenting flowers with petals, anthers and filaments were recognized as “flowering” according to [2]. The flowering date of a species was the moment when it was in flower in all plots. Species flowering only in a part of the plots were not taken into account until further survey. Flowering phenology was chronologically arranged by Julian day. Thus, we started flowering records from January 1^st^ when no plant species was in flower and annuals had just begun their life cycle. The sequence of flowering hence represents a gradient from species flowering early during the moist period to late flowering species, flowering during the dry period (three outlier species: persisting the dry period and flowering after; see below for treatment of these species). The flowering variable is hence linear. Other linear definitions would be possible, but led to consistent results.

### Sampling and trait measurements

#### 1. Vegetative traits permitting to avoid or tolerate water stress

**Presentation of species traits and their biological interest.** We chose leaf-height-seed (L-H-S) scheme that describes species’ ecological strategies [37]. Specific leaf area (SLA) is the one-sided area of fresh leaf, divided by its oven-dry mass [38]. It is strongly related to photosynthetic capacity and plant growth [37, 39]. Maximum plant height (*H*_*max*_), the distance between the top of the photosynthetic tissues and the ground level, is related to competitive ability, the degree of exposure to environmental stress such as dry atmosphere, and constitutes a proxy for the extant of seed dispersal [37, 38]. Seed mass, the dried mass of the seed, reflects plants’ capacity to avoid abiotic harshness [12].

To this L-H-S scheme we added plant life span, root depth and leaf dry matter content. A long life span indicates persistence under environmental stress [38]. Plants are characterized as annuals or perennials according to [40]. Root depth, especially deep root system, allows to access deeper soil water and hence to persist under drought stress [10]. Leaf dry matter content (LDMC), i.e. the oven dry mass of a leaf, divided by its water-saturated fresh mass [38], reflects plant growth rate and carbon storage and assimilation [41] and tends to increase under drought.

**Measurement protocol.** For every given established adult species, samples were collected when the species was in flower in all plots, or, for seeds, when the species was mature in all plots. Root depth, *H*_*ma*x_, SLA, LDMC and seed mass were measured following procedures in [38] for all 104 species and for 5 individuals of each species sampled randomly in the field, but outside the plots. Root depth and *H*_*ma*x_ were measured with a ruler, and they were expressed in m. Rooting depth corresponds to the maximum soil depth from which resources can be acquired [38]. For shallow-rooted species (approximately < 15 cm), we excavated the entire plant. For deep-rooted species, we dug a pit and described a cross-section of the soil from one face of the pit. We collected 1–5 leaves per individual and immediately stored them into a sealed bag submerged with deionised water and stored in dark and cool box, until further processing in the laboratory. Measurements were carried out the day of sampling. In the laboratory, each leaf is gently patted dry before measurements. The leaf was separated from the limbe and the rachids from the leaflets, and leaf was weighed in g. In the case of the Poaceae species, leaf sheath was separated from the leaf blade. Leaves were scanned as a computer image and the area was measured in mm^2^ using a freely downloadable software [42]. After the area measurement, each leaf sample was dried in the oven at 80°C for 48h and then was weighed with an electronic balance (10^-4^g accuracy). Seeds were collected from each plant species with an average number of 50 to 220 seeds per species. They were subsequently dried and weighed as leaves.

#### 2- Floral traits

**Presentation of species traits and their biological interest.** Floral traits reflect the adaptation of flowers to a particular pollinator and correspond to pollination syndromes [25, 26]. For each species studied, the shape of the flower, its colour and the depth were measured. Floral shape and colour are essential in determining the attraction of animals, their access to nectar, and their efficiency as pollinators [32, 43]. Flower tube depth precisely predicts the proboscis length of the insect visitors [6, 27], and nectar concentration [43]. Flowers are divided into shape categories, based on those defined by [43]. The categories identified on flower shape were dish-shaped flowers (e.g., in some Asteraceae, Euphorbiaceae, Apiaceae), gullet-shaped flowers (e.g., in some Brassicaceae, Lamiaceae), flag-shaped flowers (e.g., in some Fabaceae), and tube-shaped flowers (e.g., in some Iridaceae) or without obvious floral attractants (Poaceae). Flowers are also categorized by colour (to the human eye), following [22]. Only the corolla colour was taken into account. Colors were yellow (including yellow, whitish yellow and greenish yellow), blue, lilac, white, purple, and red (including red and reddish orange) or without obvious floral attractants (Poaceae). Colour to the human eye is only a rough proxy of colour to the pollinator’s eye, but in a comparison across all plants, flower colours that are different to the human eye are different also to the pollinator’s eye, and flower colours that are identical to the human eye are often at least similar to the pollinator’s eye [3].

**Measurement protocol.** Flowers collected in the field were immediately stored in sealed bags inside a cool box and analyzed the day of sampling. This minimizes colour changes due to storage and transpiration [3]. Flowers which were destined to tube depth measurement were preserved in 70% ethanol inside test tubes. The flower tube depth was considered as the distance between the corolla insertion and the beginning of corolla lobes [30]. To estimate the mean of flower tube depth for each species, one flower from each of the five individuals were measured with an electronic caliper (resolution = 0.01 mm). In the absence of flower tube, the depth was scored as 0 mm [27], e.g., *Euphorbia falcata* L., *Aizoon hispanicum* L. and *Herniaria fontanesii* J. Gay. Grasses were characterized as species without obvious floral attractants.

### Statistical analyses

We used general regression models (GRM) to determine the effect of plant functional traits in explaining species’ flowering phenology. GRM is used to analyze designs with both categorical and continuous predictor variables, where categorical variables are transformed into multiple binary “dummy” variables. Thus, flowering period of each species was defined as the dependent variable and the functional traits as independent variables. Our independent variables included continuous predictor variables: (1) *H*_*ma*x_, (2) root depth, (3) SLA, (4) LDMC, (5) seed mass and (6) flower tube depth, and categorical predictor variables: (7) life span, (8) flower shape and (9) flower colour of each species were included in the model. Effects for categorical predictor variables are coded in the design matrix using sigma-restricted parameterization.

We did an initial GRM analysis in order to determine significant variables using the best subset procedure with adjusted R squared as a criterion (related to AIC, but maximizing explanatory power rather than minimizing numbers of variables). Using best subset model-building technique is well-established for regression designs [44]. This technique allows finding only the “best” off all possible subsets of effects. After initial GRM analysis, we performed further GRM analyses to test for interactive effects (on phenology) of the vegetative and floral characters that had scored significant in the initial analysis. First, we did a GRM analysis by including the interaction between root depth and flower colour, again using the best subset procedure with adjusted R squared. Second, we did the same type of GRM analysis by including the interaction between root depth and flower shape. We refrained from including simultaneously both interaction terms into the same analysis (*root depth* x *flower colour*, and *root depth* x *flower shape*) as this resulted in very high multicollinearity among variables (tolerances <10%), i.e. predictor variables being highly correlated and results being unreliable. Interaction terms are uninterpretable without the raw variables included into the same model. Hence, when an interaction was retained but one of the raw variables was excluded from the model, we forced in that variable (this happened only twice and never changed the conclusion of the analysis). Some of the independent variables were log-transformed in order to normalize their residual distribution. To visualize the results of the GRMs, we calculated partial residuals (S1 Table), i.e. the residuals of phenology, accounting for the variance explained by all independent variables except the one of interest (for categorical variables partial residuals were calculated separately for each category). This also permitted to assess linearity which we could always confirm [45].

**Fig 1:**
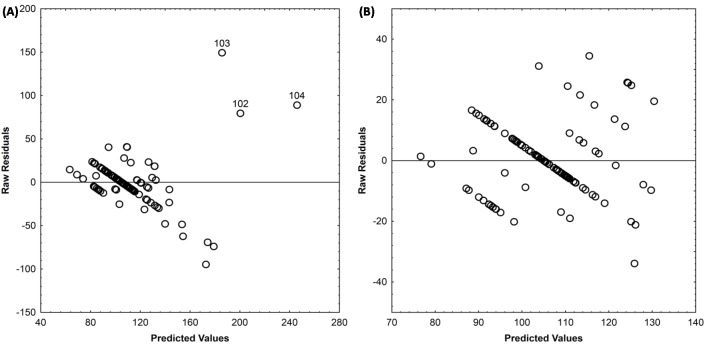
Residuals distribution (A) with outlier species (B) without outlier species.

The predicted versus residuals plot shows that three species flowering in autumn were outliers, given that all the other species flower in spring (Fig 1A). The outlier species were: *Noaea mucronata* (Forsk.) Asch. et Schw. flowering in the dry season, *Artemisia campestris* L. and *Artemisa herba alba* flower after the dry season. After excluding the three outliers, no residuals outliers remained (Fig 1B). Overall, these three species were massive statistical outliers, but biologically informative. We hence decided to present analyses with and without these outliers.

Species are phylogenetically non-independent and ideally such non-independence should be accounted for (but see [46]). At present no phylogeny is available for the flora of Algeria, and we hence had to use taxonomy as a surrogate. We considered the family level as many of our traits are conserved at this level (e.g. [47]) and as we often had no replicate species within genera. We used simple ANOVAs to test whether and which families explained our dependent variable. We found that three families had a significant effect: Amaranthaceae, Asteraceae, and Brassicaceae. To control for the effect of family, we included in the above analyses a variable with four categories corresponding to the three families and “others”.

All statistical analyses were performed in STATISTICA 10.0.

## Results

### Effect of family membership on flowering phenology

**Fig 2:**
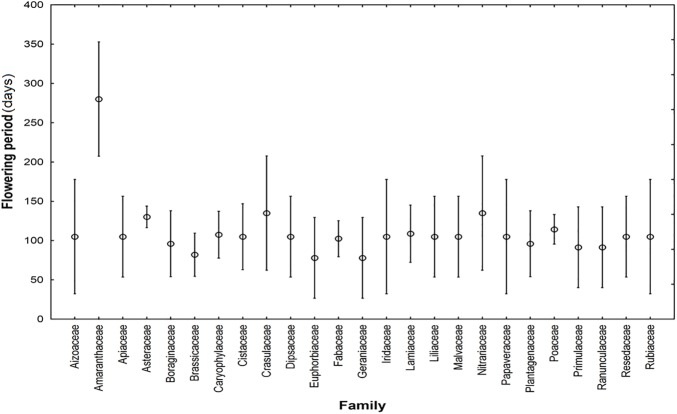
Effect of families (F_24,79_ = 1.7015, *p*<0.05) on flowering phenology. Means and 95% confidence limits are given.

One hundred and four of plant species from twenty five families were recorded in this study. Results of ANOVA showed that, overall, families had a significant effect on flowering phenology (F_24,79_ = 1.7015, *p*<0.05; Fig 2, S2 Table). However, only three of these families individually affected flowering phenology: Brassicaceae flowered particularly early (*p*<0.05), Asteraceae flowered relatively late (*p*<0.05) and Amaranthaceae flowered particularly late (*p*<0.00001).

### Explaining flowering phenology by vegetative and floral traits without accounting for interactions

**Fig 3:**
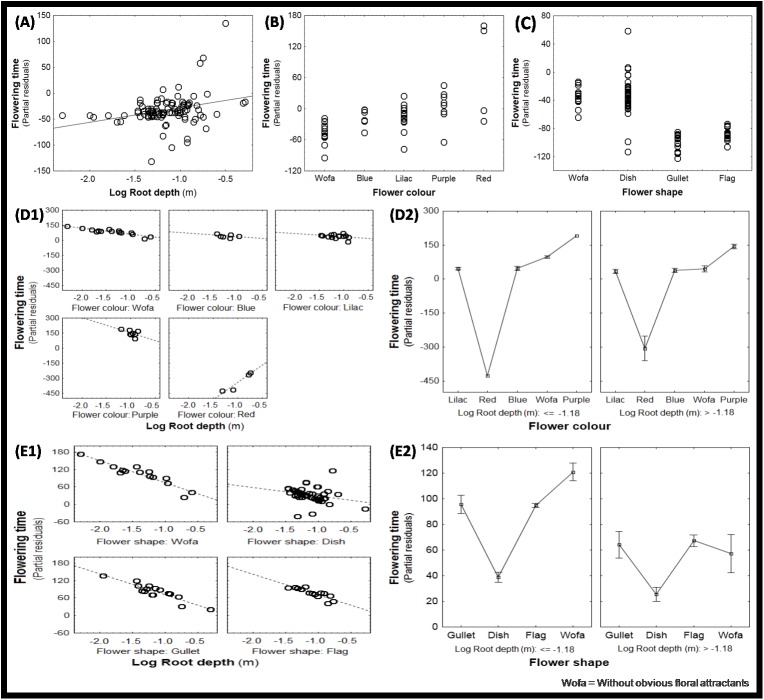
**Effect of root depth (A), flower colour (B), flower shape (C), the interactions root depth x flower colour (D), and root depth x flower shape (E) on flowering phenology.** D1 and D2 (and E1 and E2) visualize the corresponding interactions from two sides: how floral characters change the effect of root depth on phenology, and how root depth changes the effect of floral characters on phenology (using median root-depth as cut-off point). We represented partial residuals of flowering phenology, permitting to present the effect of the respective independent variable while accounting for the other variables in the model. Error bars are Standard Error (SE). Wofa = without obvious floral attractants.

General regression models accounting only for the direct effects of independent variables explained a major part of the variance: 40%–61% depending on whether or not outliers and family identity were included (Tables 1 and 2, S3 and S4 Tables). In all these models we found that avoidance traits (annual life form, large seeds) and most of the tolerance traits (low height, low SLA or high LDMC) scored non-significant or were even excluded from models. Root depth, however, delayed phenology in all models notably after excluding outlier species or accounting for family membership (e.g. see Tables 1 vs 2, S3 vs S4 Tables, Fig 3A). Flower colour (Fig 3B) and flower shape (Fig 3C) had significant effects on flowering phenology in all models, however precise results differed strongly among models used; none of the significant relationships of a given colour or shape from one model was confirmed in any of the other models (except for the accelerating effect of blue colour with and without accounting for family membership, provided that extreme residuals are excluded (see S3 vs S4 Tables). Flower-tube depth accelerated flowering phenology, but only when accounting for family membership and including residual outliers (Table 2). The most complete and balanced analysis (Model 10 in S4 Table) ranks species without attracting flower colours and such with purple colours as late flowering and such with blue flowers as early flowering.

**Table 1:** Summary of general regression models (GRM) of the effect of plant functional traits on flowering phenology, including outlier species.

Variable	Model (1)			Model (2)			Model (3)		
	Estimate	Std. Error	*P*	Estimate	Std. Error	*P*	Estimate	Std. Error	*P*
(Intercept)	159.673	14.675	*****	212.995	29.395	*****	209.868	12.734	*****
Life span: Annual	-6.279	4.226	(ns)	Excluded			Excluded		
Flower shape : Without obvious floral attractants	40.619	42.810	(ns)	243.312	160.331	(ns)	-43.743	37.630	***
Flower shape : Dish	-14.700	10.996	(ns)	-62.346	39.872	(ns)	-35.053	19.808	(*)
Flower shape : Gullet	-33.156	12.383	**	-81.737	40.102	*	-101.426	21.745	****
Flowershape : Flag	-22.564	12.520	(ns)	-69.439	39.558	(ns)	-87.356	29.134	**
Flower colour : Without obvious floral attractants	-46.522	45.752	(ns)	-327.003	184.326	(*)	-36.820	35.853	(ns)
Flower colour : Blue	-17.110	14.824	(ns)	-17.898	86.028	(ns)	-10.298	11.507	(ns)
Flower colour : Lilac	-15.557	11.401	(ns)	-13.245	49.319	(ns)	-13.357	8.999	(ns)
Flower colour : Purple	4.197	13.205	(ns)	-107.929	92.455	(ns)	3.111	10.324	(ns)
Flower colour : Red	71.084	17.211	****	447.435	58.565	*****	56.102	13.532	*****
Flower colour : Others	Excluded			Excluded			Excluded		
Log *H*_*max*_	Excluded			Excluded			Excluded		
Log Root depth	28.102	12.440	*	71.233	19.366	***	85.591	11.304	*****
SLA	Excluded			Excluded			Excluded		
LDMC	Excluded			Excluded			Excluded		
Log Seed mass	Excluded			Excluded			Excluded		
Log Flower tube depth	Excluded			Excluded			Excluded		
Log Root depth x Flower colour : Without obvious floral attractants	NA	NA	NA	-60.421	22.644	**	NA	NA	NA
Log Root depth x Flower colour : Blue	NA	NA	NA	-35.767	59.840	(ns)	NA	NA	NA
Log Root depth x Flower colour : Lilac	NA	NA	NA	-34.370	36.589	(ns)	NA	NA	NA
Log Root depth x Flower colour : Purple	NA	NA	NA	-152.689	78.432	(ns)	NA	NA	NA
Log Root depth x Flower colour : Red	NA	NA	NA	341.654	48.029	*****	NA	NA	NA
Log Root depth x Flower shape : Without obvious floral attractants	NA	NA	NA	NA	NA	NA	-74.779	15.305	*****
Log Root depth x Flower shape : Dish	NA	NA	NA	NA	NA	NA	-28.708	15.896	(*)
Log Root depth x Flower shape: Gullet	NA	NA	NA	NA	NA	NA	-71.199	17.041	****
Log Root depth x Flower colour : Flag	NA	NA	NA	NA	NA	NA	-7.752	26.501	**

**Model (1):** GRM without interaction terms, R^2^ = 0.41%, df = 92; **Model (2):** GRM with the interaction between root depth and flower colour, R^2^ = 0.61%, df = 87; **Model (3)**: GRM with the interaction between root depth and flower shape, R^2^ = 0.64%, df = 89. See S3 Table for analyses without outlier species.

(*): *P*<0.10.

*: *P*<0.05.

** *P*<0.01.

***: *P*<0.001.

****: *P*<0.0001.

*****: *P*<0.00001.

(ns): Not significant.

NA: Not applicable. Excluded = variables not retained by best subset search. Estimate = estimate of regression parameter.

**Table 2:** Summary of general regression models (GRM) of the effect of plant functional traits and—contrary to Table 1—family membership on flowering phenology, including outlier species.

Variable	Model (4)			(Model (5)			Model (6)		
	Estimate	Std. Error	*P*	Estimate	Std. Error	*P*	Estimate	Std. Error	*P*
(Intercept)	214.024	16.805	*****	233.282	27.231	*****	253.224	10.283	*****
Family: Others	-39.4198	8.572	****	-16.512	8.513	(*)	-40.153	5.280	*****
Family: Amaranthaceae	98.0073	22.185	****	31.767	22.353	(ns)	120.074	13.793	*****
Family: Asteraceae	10.0736	9.591	(ns)	26.980	8.669	**	-7.163	6.678	(ns)
Life span: Annual	-5.3272	3.656	(ns)	-3.638	3.155	(ns)	Excluded		
Flower shape : Without obvious floral attractants	4.2756	38.269	(ns)	147.793	143.259	(ns)	-83.767	14.680	*****
Flower shape : Dish	-31.3662	10.511	**	-59.451	35.184	(ns)	-49.147	13.407	***
Flower shape : Gullet	-8.0771	11.956	(ns)	-41.943	35.927	(ns)	-80.826	14.659	*****
Flowershape : Flag	-9.6064	11.267	(ns)	-43.007	35.598	(ns)	-57.042	22.121	*
Flower colour : Without obvious floral attractants	-18.8523	40.941	(ns)	-241.618	164.851	(*)	Excluded		
Flower colour : Blue	-15.9414	12.878	(ns)	6.747	77.181	(ns)	Excluded		
Flower colour : Lilac	-2.6035	9.974	(ns)	-10.052	43.290	(ns)	Excluded		
Flower colour : Purple	14.2277	13.420	(ns)	-97.640	80.514	(ns)	Excluded		
Flower colour : Red	23.2500	16.321	(ns)	337.443	64.575	*****	Excluded		
Flower colour : Others	Excluded			Excluded			Excluded		
Log *H*_*max*_	14.6911	10.950	(ns)	13.619	9.551	(ns)	Excluded		
Log Root depth	24.4423	11.423	*	59.148	18.869	**	89.112	8.233	*****
SLA	0.0106	0.008	(ns)	0.008	0.007	(ns)	Excluded		
LDMC	Excluded			Excluded			Excluded		
Log Seed mass	Excluded			Excluded			Excluded		
Log Flower tube depth	-36.6461	11.189	**	-30.079	10.007	**	-21.461	6.621	**
Log Root depth x Flower colour : Without obvious floral attractants	NA	NA	NA	-60.887	20.218	**	NA	NA	NA
Log Root depth x Flower colour : Blue	NA	NA	NA	-6.181	54.188	(ns)	NA	NA	NA
Log Root depth x Flower colour : Lilac	NA	NA	NA	-28.940	32.438	(ns)	NA	NA	NA
Log Root depth x Flower colour : Purple	NA	NA	NA	-133.452	68.420	(ns)	NA	NA	NA
Log Root depth x Flower colour : Red	NA	NA	NA	269.866	49.130	*****	NA	NA	NA
Log Root depth x Flower shape : Without obvious floral attractants	NA	NA	NA	NA	NA	NA	-78.300	11.233	*****
Log Root depth x Flower shape : Dish	NA	NA	NA	NA	NA	NA	-32.500	12.300	**
Log Root depth x Flower shape: Gullet	NA	NA	NA	NA	NA	NA	-72.182	12.587	*****
Log Root depth x Flower colour : Flag	NA	NA	NA	NA	NA	NA	-49.501	19.810	*

**Model (4)**: GRM without interaction between significant variables, R^2^ = 0.61%, df = 86; **Model (5)**: GRM with the interaction between root depth and flower colour, R^2^ = 0.72%, df = 81; **Model (6)**: GRM with the interaction between root depth and flower shape, R^2^ = 0.80%, df = 90. See S4 Table for analyses without outlier species.

(*): *P*<0.10.

*: *P*<0.05.

** *P*<0.01.

***: *P*<0.001.

****: *P*<0.0001.

*****: *P*<0.00001.

(ns): Not significant.

NA: Not applicable. Excluded = variables not retained by best subset search. Estimate = estimate of regression parameter.

### Explaining flowering phenology by vegetative and floral traits accounting for interactions

Here, we conducted general regression models accounting for the direct effect of independent variables as well as for the interaction terms among the vegetative trait (root depth) and the floral traits (flower colour and flower shape) that had scored significant in the above analyses. Looking at the analyses including the root depth x flower colour interaction term, we found that its inclusion increased the variance by 5–20% (average 11%). We consistently found in all four analyses (including or not family membership and extreme residuals) that deep-rooted species had later phenologies. We consistently found in all except two analyses that deep roots accelerated the flowering of species that have no attracting colours and delayed that of species with red flowers (negative and positive interaction terms, respectively; models 2 and 5 in Tables 1 and 2). In the remaining analyses (without extreme outliers and with family membership, S3 and S4 Tables) we found that deep roots retarded flowering of purple flowers. Consistently, Fig 3D1 shows that the opposing shifts of phenology with root depth in different flower colours lead to a phenological convergence among colours with increased root depth. Equally consistently, Fig 3D2 shows that with increasing root depth flowering dates converged among different flower colours.

Looking at the analyses including the root depth x flower shape interaction term, we found the variance increases by 6–23% compared to analyses without interaction term (mean = 13%). We found that species with deep roots tend to delay flowering. In the analysis with extreme residuals (including family membership or not), we found that deep roots accelerated flowering notably of species without attractive flower shapes or with gullet-shaped flowers, to a lesser degree of species with flag-shaped flowers and to the lowest degree dish-shaped flowers (Tables 1 and 2). This ranking of interaction terms is basically consistent with that in the analysis without extreme residuals (including family membership or not): the interaction root depth x dish shaped is the least negative–in fact it is even positive. Dish-shaped flowers flowered relatively early in species with shallow roots, but this effect disappeared in deep-rooted species (see Models 9 and 12 in S3 and S4 Tables). Consistently, Fig 3E1 shows that in dish-shaped flowers, flowering was exceptionally early only in shallow-rooted species. Equally consistently, Fig 3E2 shows that with increasing root depth, flowering dates converge among different flower shapes.

Overall, the interaction terms suggest that an effect (of a given floral colour or floral shape) that is present among shallow-rooted species disappears among deep-rooted species. This result is consistent with an overall decline of variability of phenology among floral characters when comparing shallow-rooted to deep-rooted species: ANOVAS of effects of floral colour and shape on phenology give F = 6.9 and 3.9 for species of below-median root-depth, but only 3.8 and 2.7 for species of above-median root-depth. In other words, phenologies always depend on floral characters, but much less so among deep-rooted species than among shallow-rooted species. Note that this convergence of flowering phenologies among floral types in deep-rooted species does not reflect a decline in overall variability of phenologies as such. In fact, flowering phenologies are distinctly more variable among deep-rooted species than among shallow-rooted species: standard deviations of phenologies are 51.6 days among species of above-median root-depths vs only 17.4 days among species of below-median root-depths; the corresponding coefficients of variation are 0.42% vs only 0.17%.

## Discussion

### Vulnerability to drought stress constrains flowering phenology

In this study, we have shown that plants flowering later are those that develop deep roots allowing them to access deeper soil water during the dry season [10, 14, 15]. Deep roots, moreover, allow plants to store significant quantities of water in their soft tissues, and therefore maintain activity during the dry season [16]. Remaining active during the dry season may be also due to the higher amount of soluble sugar and proline accumulated in roots [48]. These osmolytes, used as a biochemical marker of drought stress [48], can induce an osmotic adjustment of roots and allow plants to maintain water uptake during the dry season [17]. Thus, remaining physiologically active during the dry season, allows plant species to flower during the dry season, i.e. *Noaea mucronata*, or after the dry season, i.e. *Artemisia campestris* and *Artemisa herba alba* [21]. Flowering at these periods has been suggested to be a case of temporal niche partitioning [49] that allows plants to reduce competition [50] and hence facilitate species co-existence [5]. This finding leads to the idea that in semiarid steppes, deep roots may be advantageous for plants not only to survive drought stress, but also to flower later and escape competition for pollinators.

Flowering phenology was largely independent of most functional traits. Thus, tolerance traits operating at the level of leaves or height do not relax flowering phenology, nor do traits related to avoidance. Initially, we expected that such traits have important effects on phenology as it has been reported that leaf traits are key variables implicated in plant functional ecology [51]. Indeed, in sub-Mediterranean climate where water stress is comparatively less severe, leaf traits are considered as a stress-tolerance mechanism. Particular leaf anatomies permit to protect plants from water stress, through adaptations to limit evapotranspiration (scleromorphic leaves) and retain water reserves (succulent leaves) [50]. However, these factors might constrain only species of short life span, and only such species might profit from tolerance traits. Perennial species, in contrast, have generally accumulated resources during precedent years [2] allowing to flower at any moment irrespective of seed size or height or leaf traits. Perennials hence do not flower or produce until they have accomplished enough vegetative growth [11]. Overall, perennial growth might explain why phenology did not depend on avoidance or tolerance traits, other than root depth.

### Pollinator types constrain flowering phenology

Different floral traits attract different insect pollinators [22, 27, 29], and pollinator composition and richness shift among seasons thereby potentially constraining phenology of plants species with a given floral trait. For instance, [31] showed that, in Mediterranean regions, honey bees may be the most abundant flower visitor in April and notably (86% to 90.9%) of “gullet” and “flag” shaped flowers [31]. In our study, these flowers are found in Brassicaceae and Fabaceae and indeed flower in early spring. Honey bees also appeared to prefer blue flowers that flowered early (at least in the analyses without extreme residuals and with family membership). Furthermore, beetles, true bugs and notably ants visit open flowers, i.e. dish-shaped flowers [31] probably due to easily accessible nectar [43]. Ants remain active during the dry season and dish-shaped flowers might hence be expected to still flower during the dry season. In our analyses, the effect of dish shape on phenology scored differently in different analyses, but the most complete and balanced analysis (model 12 including family membership and interaction terms with flower shape and excluding extreme residuals) showed that dish shape strongly delayed flowering phenology into the warm and the dry season. We may hence speculate that future climatic warming might affect the abundance and/or the activity of pollinators, suggesting that plant-pollinator network and ecosystem processes and services would be altered [52]. Such a shift in pollinator phenology may lead to a shift in phenology of plants with the associated floral types.

### Response to drought stress controls plant-pollinator interactions

We found that vulnerability to abiotic harshness due to shallow roots controlled the effect of floral characters on flowering phenology. Specifically, in shallow-rooted species floral characters influenced flowering phenology more than in deep-rooted species. This observation is inconsistent with our hypothesis that reduced activity of shallow-rooted species during the dry season might prevent them from adjusting flowering phenology to dry season pollinators. In contrast, the observation that in shallow-rooted species floral characters influence flowering phenology more than in deep-rooted species is consistent with our alternative hypothesis: shallow-rooted species might be more water-stressed and might be constrained to invest water and carbon into flowering only during the period when their respective pollinators are most active. Thus, shallow-rooted species with floral traits attracting different pollinators strongly adjust their flowering phenologies to the different phenologies of their respective pollinators. This would imply that performance under abiotic stress like drought might not be traded off against performance in biotic interactions with pollinators. Simply, abiotically performant, deep-rooted species have more water and carbon to invest into biotic interactions and might be less constrained in flowing phenology.

### Effect of taxonomic position on flowering phenology

Accounting for family membership often distinctly increased R^2^ and several families were significant predictors of flowering phenology. Divergence in flowering phenology among families might be associated with divergences in functional traits [2], and through a common descent the closely related species within families often exhibit more similar functional traits than distantly related species among families [53]. As a consequence, species within families tend to flower at similar times, i.e. flowering phenology is phylogenetically conserved (as already demonstrated by [2, 30, 54]). Interestingly, after accounting for family membership, floral colour and floral shape as such often remain significant predictors of phenology, but the precise colour or shape and even the sign of relationships changed. Variation of phenologies across families and variation within families might hence be controlled by partly different processes. Other results, in contrast, appear to be consistent with and without accounting for families, notably those on vegetative traits and how they interact with floral traits to control flowering phenology. The processes associated with these traits–vulnerability to drought and how it constrains plant-pollinator interactions–might hence be consistent across many families.

## Conclusion

Our findings suggest three main implications. First, plant adaptations to water stress may control the seasonal availability of flowers to pollinators. Second, root characters are essential to understand flowering phenologies. Such root traits are only rarely considered in studies covering large number of species. In fact, our trait database providing belowground and aboveground vegetative traits, floral traits and phenology of 104 species may be the first of its type for a North African steppe. Third, the degree to which a plant’s interaction with a given pollinator type constrains the plant’s flowering phenology may depend on the plant’s tolerance to abiotic constraints. Abiotically less tolerant (shallow-rooted) species are more constrained by floral traits, suggesting that plants suffering higher abiotic stress may be forced to limit flowering to the activity peaks of their respective pollinators. This hypothesis requires experimental testing within species in the future, manipulating both stress and pollinator availability. Moreover, further studies are required (i) to determine pollinator species that constrain flowering phenology, (ii) to explain why multiple of the functional traits we tested do not affect flowering phenology and (iii) to elucidate the phylogenetic constraints on flowering phenologies.

## Supporting information

S1 Table Partial residuals of flowering time calculated to present effects of functional traits on flowering phenology. (XLSX)

S2 Table Flowering phenology of different families. Mean and Standard Error (SE) at 95% are given.
(XLSX)

S3 Table Summary of general regression models (GRM) of the effect plant functional traits on flowering phenology: without outlier species. Model (7): GRM without interaction between significant variables,R^2^ = 0.40%, df = 89; Model (8): GRM with the interaction between root depth and flower colour, R^2^ = 0.45%, df = 83; Model (9): GRM with the interaction between root depth and flower shape, R^2^ = 0.45%, df = 84. (*): *P*<0.10; *: *P*<0.05; **: *P*<0.01; ***: *P*<0.001; *****: *P*<0.00001; (ns): Not significant; NA: Not applicable. Excluded = variables not retained by best subset search. Estimate = estimate of regression parameter.
(XLSX)

S4 Table Summary of general regression models (GRM) of the effect of plant functional traits—contrary to S3 Table—and family membership on flowering phenology: without outlier species. Model (10): GRM without interaction between significant variables, R^2^ = 0.46%, df = 89; Model (11): GRM with the interaction between root depth and flower colour, R^2^ = 0.54%, df = 48; Model (12): GRM with the interaction between root depth and flower shape, R^2^ = 0.50%, df = 82. (*): *P*<0.10; *: *P*<0.05; ** P<0.01; ***: *P*<0.001; *****: *P*<0.00001; (ns): Not significant; NA: Not applicable. Excluded = variables not retained by best subset search. Estimate = estimate of regression parameter.
(XLSX)
